# Melanophore and fluoroleucophore photo-protect the Arabian killifish, *Aphanius dispar*, embryo from ultraviolet light

**DOI:** 10.1038/s41598-026-37311-6

**Published:** 2026-02-03

**Authors:** Maryam Alenize, Rashid Minhas, Tetsuhiro Kudoh

**Affiliations:** 1 Biosciences, University of Exeter, Exeter, EX4 4QD UK; 2 MRC Centre for Medical Mycology, University of Exeter, Exeter, EX4 4QD UK; 3 Department of Biology, Faculty of Science, University of Tabuk, 71491 Tabuk, Saudi Arabia

**Keywords:** Arabian killifish, Pigments, UV, UVC, Fluoroleucophore, Melanophore, Photoprotection, Developmental biology, Ecology

## Abstract

Pigment cells in fish species play crucial roles in forming colour patterns of each species and other physiological characteristics, including photoprotection. Research on animal pigment cells’ photoprotection has primarily concentrated on black pigment cells, known as melanophores. However, the roles of other pigment cells on UV protection remain poorly understood. In addition, UV sensitivity/resistance mechanisms have been studied in a small number of selected model organisms, and difference of UV sensitivity has not been fully investigated in different species. In this study, we use the Arabian killifish embryos as a novel model for studying the mechanisms of UV protection by different pigment cells. This species features highly fluorescent pigment cells called fluoroleucophores and black pigment cells melanophores. The fluorescent pteridine pigments and black melanin pigments are generated by genes *gch* (GTP cyclohydrolase) and *tyr* (tyrosinase), respectively. We generated *gch-/-* and *gch-/-tyr-/-* double mutant lines using CRISPR/Cas9 genome editing and examined the UV sensitivity of WT and these mutant embryos. Firstly, we have observed that Arabian killifish embryos showed higher UV resistance to the one in zebrafish from which majority of UV toxicity studies was conducted in fish species so far. Secondly, both morphology and gene expression data revealed that the *gch-/-tyr-/-* double mutant line exhibited the highest UV sensitivity, and the *gch-/-* line also demonstrated a greater stress response compared to wild type (WT). The study revealed a diversity of UV resistance between fish species and also identified the roles of black and fluorescent pigment cells in providing effective UV protection from the early stages of embryonic development.

**Supplementary Information:**

The online version contains supplementary material available at 10.1038/s41598-026-37311-6.

## Introduction

Pigment cells in fish play a crucial role in determining the colour and structure of their skin, scales, and other body parts, with speciation closely linked to these colour patterns^1^. These diverse skin colour patterns serve multiple functions, including camouflage for predator avoidance, social signalling, mate evaluation and photo-protection^2,3^.

Some pigment cells^4,5^, like melanophores, xanthophores, and erythrophores, absorb light, while others, such as iridophores and leucophores, reflect light^6,7^. In teleosts, melanophores, iridophores, and xanthophores are widely distributed in many fish species, whereas leucophores are relatively rare^5,6,8,9^.

Melanophores are dendritic cells that align parallel to the skin’s layers and contain pigmented organelles known as melanosomes. These melanosomes house melanin, the dark pigment responsible for the brown or black colouration in fish. The primary function of the melanophore is to synthesise, disperse or aggregate melanin within the cytoplasm, enabling the fish to change colour and providing protection from UV radiation^10^.

Leucophores contain two types of pigment molecules: a weakly fluorescent pteridine pigment and a white pigment composed of urea^6,11^. Their reflective properties may also assist fish in maintaining an optimal body temperature by reflecting sunlight^5,6,8,9,12^.

Fluoroleucophores, found in the Arabian killifish, are closely related to leucophores but exhibit greater fluorescence than those reported in other teleost fish species^12,13^. We have previously found that GTP cyclohydrolase (*gch*) morphant and crispant show loss of fluorescent pigment from the Arabian killifish indicating the crucial role of *gch* in generating the fluorescent pigment related to the group of pteridine molecules^13^.

Arabian killifish, *Aphanius dispar*, inhabits both freshwater and marine habitats and is widely distributed across the Middle East, from Western India and the Arabian Peninsula to West Africa. This species is commonly found in estuaries, lakes, and coastal areas, often residing in shallow waters, streams, and rocky habitats where it spawns in rock crevices^14,15^. *A. dispar* is euryhaline, capable of tolerating a wide range of salinities, from freshwater to brackish and seawater^16^. Arabian killifish often inhabit shallow waters in Middle Eastern rivers and seas, where vegetation is scarce, and both embryos and adult fish are exposed to intense sunlight. Akbarzadeh et al. 2014 reported that *A. dispar* can endure elevated temperatures, surviving up to 40 °C^17^. The presence of pigment cells developing from an early stage of the embryonic stage^13,18^ suggests the potential importance of such pigment cells for light protection in the habitats^13^.

The sun is known to be the primary source of all forms of ultraviolet (UV) rays reaching the Earth’s surface. Ultraviolet radiation is divided into three major types based on the wavelength range measured in nanometres (nm): ultraviolet A (320–400 nm) has the longest wavelength with relatively harmless and moderate energy, Ultraviolet B (280–320 nm), with moderate harmful effects, and ultraviolet C the shortest wavelength (200–280 nm) with severe dangers^19,20^. All these UV lights can cause DNA damage and oxidative stress of proteins and lipids in the cell with different extent of severity. UVA consists of up to 95% of the ultraviolet that reaches the earth’s surface, and it can penetrate the water column. UVA and UVB can reach to the ground and shallow water, but UVC are mostly filtered and absorbed by ozone, oxygen, and water within the atmospheric layer^21,22^. It has been shown that fish embryos are highly sensitive to UV light; exposure to UV radiation showed increased embryo malformations associated with decreased survival^23–29^. Besides deleterious effects, UV can also activate DNA repair pathway^23,30,31^, therefore mixed effects with less toxic UVA and toxic UVB/C can be highly complicated.

Though there are many pigment cell types in the fish species, our knowledge in the roles of these cells other than melanophore in UV protection is very limited. We have previously observed that in the Arabian killifish embryos, melanophore, fluoroleucophores and iridophores are neatly piled up as a unit with layer structure having iridophore at the skin surface, leucophore middle and melanophore in the deep layer suggesting roles of these pigment cells and associated pigments in photoprotection^13^. In this report, we used Arabian killifish embryos as a model to study UV toxicity. We utilised CRISPR/Cas9 genome editing in Arabian killifish embryos to generate two specific pigment mutant lines: a *gch* mutant defective in fluorescent pteridine pigment synthesis in the fluoroleucophore and a *gch/tyr* double mutant which is devoid of melanin synthesis in the melanophores besides the *gch* mutation. We quantitatively analysed toxicity of UVC 254 nm and stress response using morphological and gene expression analyses. Our data suggests the role of melanophore and fluoroleucophore in UV protection, and physiological role of multiple pigment cells for the survival of fish in environments with intense sunlight.

## Materials and methods

### Arabian killifish husbandry and embryos collection

The Arabian killifish *Aphanius dispar* wild type, *gch*^−^/^−^ and *gch-/-/tyr-/-* lines, were sourced and maintained in the Aquatic Resource Centre at the University of Exeter. These fish were kept in a recirculation system under artificial photoperiods of 14 h of light to 10 h of darkness. The Arabian killifish *A. dispar* eggs were collected in egg spawning chambers and stored in Petri plates at a temperature of 28 °C and a salinity of 35 ppt.

### Genome editing of ***gch*** and ***tyr*** gene by CRISPR/Cas9

To generate *gch* mutant and *gch/tyr* double mutant, two each CRISPR RNAs (crRNAs) were designed from the cDNAs of these two genes (Genbank accession numbers, PQ588425 for *gch* and PQ588426 for *tyr* respectively). The designed crRNA sequences for these genes were: *gch*_crRNA1 GTCCCGCTTACCCGCTCTGG and *gch*_crRNA2 GAGGAACTGAATGGCCTTGG), *tyr*_crRNA1 CTGGTCTCAGATGAACCCAA and *tyr*_crRNA2 AGTGTGCACTGATAATCTGA. Firstly, the mixture of two *gch*_crRNAs at 50 ng/µl each, 100 ng/µl tracrRNA and 200 nM Cas9 nuclease (NEB) was injected into the one-cell stage WT eggs with approximately 1nl volume. Embryos which have completely lost fluorescence were selected (88%) and raised to adult (*gch*^*−*^*/*^*−*^ F0 generation). The *gch* mutant F1 line was established from natural spawning. Eggs were collected from these F1 fish and injected with mixture of two *tyr*_crRNAs to generate *gch/tyr* double mutant. All embryos which have completely lost the black pigment were selected (83%) and raised to adult (*gch-/-/tyr -/-* F0).

### DNA extraction, PCR and genotyping

For confirming the mutation in the *gch* and *tyr* genes, genomic DNA was extracted from the F1 generation of *gch/tyr* double mutant embryos were collected^32^. Each embryo was snap frozen in liquid nitrogen, and subsequently dissolved in 50ul of 50mM NaOH and homogenised using an electronic pestle, incubated at 95 °C for 10 min, next, cooled down the mixture to 16 °C. Then, 5ul of 1 M of Tris hydrochloride (1 M Tris–Hcl, pH = 8.0) was added.

The targeted genomic region of *gch* or *tyr* gene was amplified by using Promega PCR Master Mix Green with using following volumes: 1 µl of genomic DNA mixture, 1 µl each forward and reverse primers (25 µM): 12.5 Promega PCR Master Mix Green and nuclease free water to 25 µl PCR reaction. Primer sequences flanking *gch* targeted region were: *gch*_F (AAAAGTTGGAGAAACCGCCG), *gch*_R (GATGGTCTCATGGTAGCCCT), *tyr*_F (GAAGGAGATGGCTCACCT), *tyr*_R (GCTAATGAGGTTGGGATT). The amplification conditions were as follow: 95 °C 1 min, 35 cycles (95 °C 30 s, 58 °C 1 min, 72 °C 1 min) with the PCR machine 2720 Thermal Cycler (Applied Biosystems). The final extension step was performed at 72 °C for 5 min. The PCR product was purified by Promega PCR clean-up system and cloned into a pGEM-T Easy Vector for sequencing.

### UVC radiation exposure

UVC radiation was provided by a UV crosslinker (Analytic Jena US, UVP Cross linker, CL-3000) containing six LED- lamps at wavelength 254 nm operated at 250 V. Light irradiance of this machine is 1mW/cm^2^. Ten embryos at 4dpf were placed in a Petri dish containing 25 ml of 35 ppt Artificial seawater. UVC lamps were placed at 17 cm above the dish’s surface. Four conditions were set up for exposure: No UV exposure (control) 0 mJ/cm^2^, UV 25 mJ/cm^2^ (25 s), UV 50 mJ/cm^2^ (50 s) and UV 100 mJ/cm^2^ (100 s), After UV irradiation, all exposed and non-exposed embryos were transferred to an incubator at 28 °C for 24 h with 12 h light and 12 h dark.

### Mortality, survival, heat beat rates and malformation

The effects of UVC exposure on the morphology and development of embryos were examined at 5 days post fertilization (5 dpf) until the hatching, following 24 h of UV exposure. Mortality and malformation were analysed daily, with immediate removal of any dead embryos to avoid contamination. The mortality was conducted by observing locomotion, heartbeat and blood circulation using a Nikon SMZ1500 and an Olympus SZX16 microscope. The heart-beat rates of treated and untreated UV of *A. dispar* were monitored at 24-, 48- and 96-hours post-UV exposure. The heartbeats of all 10 embryos were observed for 30 s, and then the heart rate per minute was calculated. Before manual counting, embryos were kept at room temperature for 10 min to allow the heartbeat to stabilize. The malformation rates for UV exposure *A. dispar* embryos were assessed through microscopic examination at 24 h post UV exposure until the hatching day and imaging for the main body changes. Pigment cell aggregation was observed microscopically in the designated regions. Every experiment was performed independently in triplicate.

### Imaging

For imaging purposes, the embryos were immersed in gel canals by leaving 1% agarose (sigma, A9539) made of deionised water to set on 1.2 mm diameter glass tubes. Photographs were taken using a Nikon DS-L3 (DS-Fi2-L3u) camera and NIS-Elements software (version 4.30) on Nikon SMZ1500 microscope using normal incident light and appropriate GFP and RFP filters.

### RNA extraction, cDNA synthesis and real-time quantitative PCR (qPCR)

The total RNA was extracted from embryos after 24 h post-UV exposure from both untreated control and treated UV groups. Five embryos from each of the four conditions: Control (untreated), UV 25 mJ/cm^2^, UV 50 mJ/cm^2^, and UV 100 mJ/cm^2^, were collected from three different strains: *A. dispar* WT, *A. dispar gch*^*-*^*/*^*-*^, and *A. dispar gch-/-tyr-/-*. The collected embryos were placed in a tube containing 200 µl of Trizol reagent (RTI, Sigma T9424) and homogenized using an electric pestle. An equal volume of ethanol (95–100%) was added, and the total RNA was then isolated using a Direct- Zol TM RNA miniprep kit) as per the manufacturer’s protocol. RNA was eluted in nuclear-free water, and concentration was assessed using Qubit and Nanodrop, while RNA quality and integrity were verified using Tape Station. Subsequently, the RNA samples were stored at −20 °C.

The cDNAs were synthesised using the Thermo Scientific™ RevertAid™ kit as per manufactures’ protocol. All RNA samples were normalised to equal concentration (200 ng/ul) before setting up the reaction. Each reaction contained 1ul of RNA, 1 µl of Random hexamer primer and nuclease-free water to achieve a total volume of 12 µl for each reaction. Subsequently, 5X Reaction Buffer (4 µl), RiboLock RNase Inhibitor (20 U/µL) (1 µl), 10 mM dNTP Mix (1 µl) and of RevertAid M-MuLV RT (200 U/µL) were added then centrifuge briefly. The reaction was then incubated at 42 °C for 60 min then terminated the reaction by heating at 70 °C for 5 min. cDNA samples were stored at −20 °C to − 80 °C.

Real time PCR was performed using Luna Universal qPCR Master mix (NEB, Catalogue M3003S) in a total volume of 10 µl. Each reaction contained 5 µl of Luna mix Universal qPCR Master mix, 0.5 µl of forward and reverse primers (10 µM), 1 µl of cDNA 3 µl of NF water. qPCR was performed on QuantStudio™ 3 Real-Time PCR System with the following cycling conditions: initial denaturation at 95 °C for 1 min, followed by 35 cycles of denaturation at 95 °C for 15 s, and extension at 60 °C for 30 s.

### Ethical declaration

All experiments and methods were performed in accordance with relevant guidelines and regulations of the UK Home Office with the licence number PP4402521. All methods are reported in accordance with ARRIVE guidelines. All experiments and methods are approved by the Animal Welfare and Ethical Review Board (AWERB) at the University of Exeter (ID 9051829).

## Results

### Generation of fluorescent and black pigment mutants

To generate pigment mutant lines defective in fluorescent pigment (*gch* mutant) and both fluorescent and black pigments (*gch/tyr* double mutant), the *gch* gene was initially knocked out by injecting two crRNAs as a mixture (*gch*_crRNA1 and *gch*_crRNA2). 88% of injected embryos showed complete loss of fluorescent pigment. These non-fluorescent embryos were raised to adult to establish the F0 founders of the *gch* mutant line. Eggs were then collected from these F0 fish through natural spawning and raised to form the F1 generation.

To generate *gch/tyr* double mutant, eggs were collected by natural spawning from the *gch* mutant F1 generation and injected with two *tyr*_crRNAs (*tyr*_crRNA1 and *tyr*_crRNA2). Among these injected embryos, 83% showed a complete loss of black pigment. These embryos were raised to adult to establish *gch/tyr* double mutant founders.

**Fig. 1:**
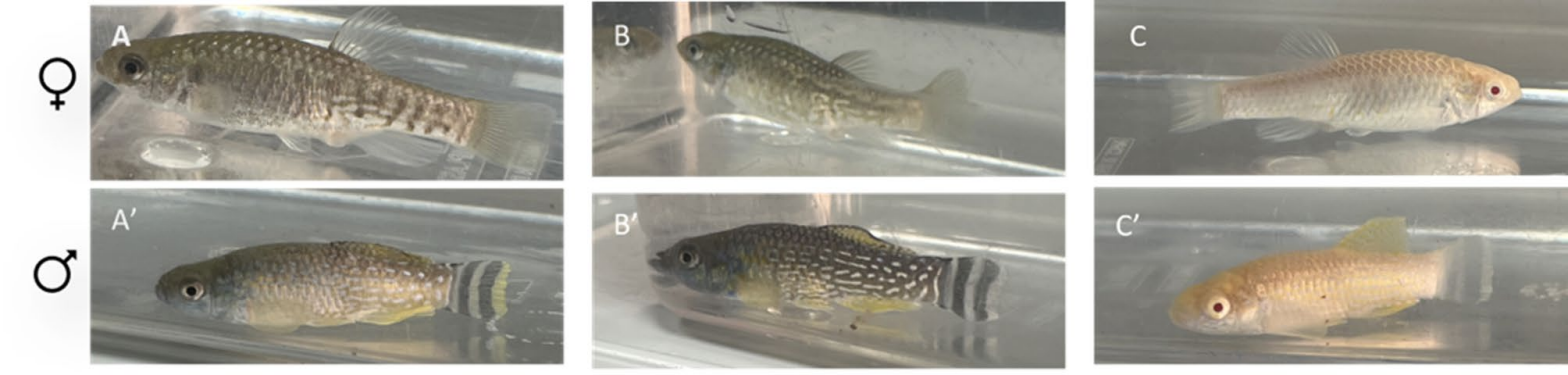
Arabian killifish *Aphanius dispar* adult fish with WT (A, A’), *gch* mutant (B, B’) and *gch/tyr* double mutant (C, C’) with females (A, B, C) and males (A’, B’, C’).

The adult *gch* mutant fish did not show any noticeable differences in colour pattern compared to wild-type fish (Fig. 1A. A’, B, B’). However, the *gch/yr* mutant fish displayed a clear loss of dark pigments and showed a typical albino colour pattern in both the skin and lens (Fig. 1A, A’ and C, C’).

**Fig. 2:**
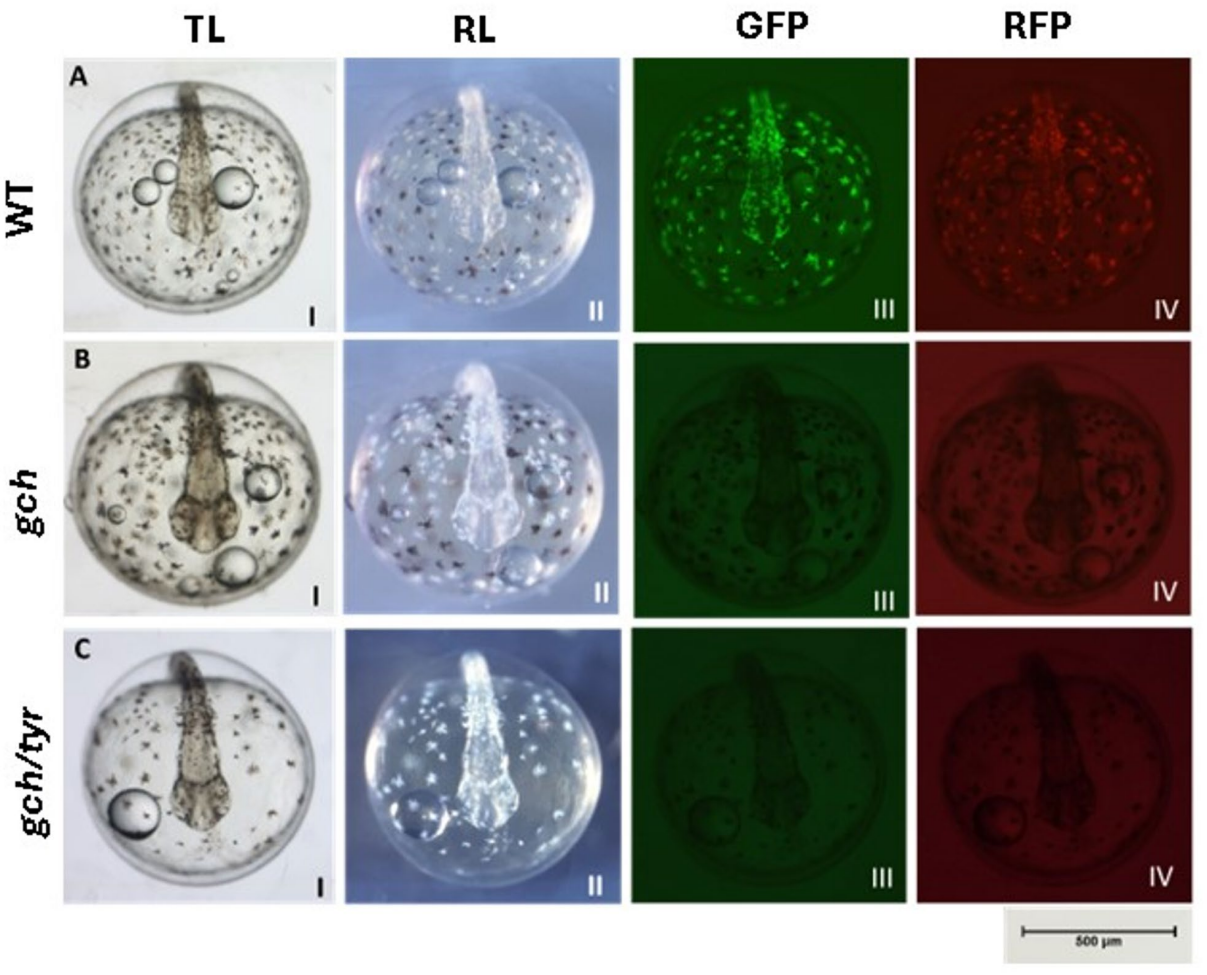
Arabian killifish ***Aphanius dispar*** whole e***m***bryos at 3 days post-fertilization from anterior dorsal view. **A** *A. dispar* wild-type WT, **B** *A*. *dispar gch-/-* (induce loss fluorescent in leucophores), **C** *A. dispar gch-/-tyr-/-* (Group image in Fig. S1). *TL* Transmission light, *RL* Reflection light, *GFP* fluorescent light with GFP filter, *RFP* Fluorescent light with RFP filter.

At the embryonic stage WT, *gch* and *gch/tyr* double mutants exhibited distinct and clear differences their colour pattern. In the normal transmission light, colour difference between the melanophore and fluoroleucophore are not clear (Fig. 2AI, BI, CII) however with the reflection light, fluoroleucophore become white and clearly distinguished with black melanophore (Fig. 2AII, BII, CIII). As reported from the morphant and crispants from Hamied et al. 2020^13^ mutations in the *gch* caused loss of fluorescence from the fluoroleucophores (Fig. 2AIII, IV, BIII, IV, CIII, IV). In addition, with the mutations of *gch/tyr*, both fluorescence and black pigments were lost (Fig. 2CII).

### Genotyping of fluorescent and black pigments mutants

**Fig. 3:**
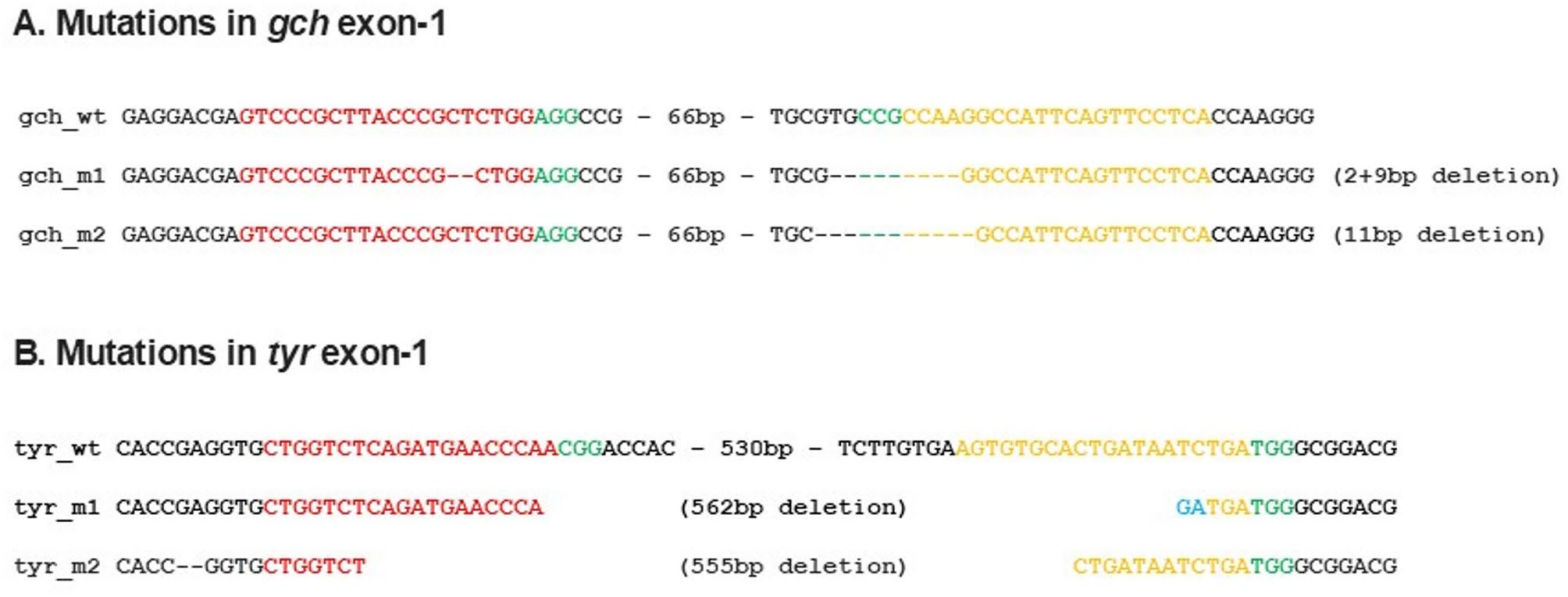
Mutations in the *gch* and *tyr* exon 1. **A** and **B** *gch* and *tyr* sequence in the exon-1 of each gene around the crRNA1 (red) and crRNA2 (orange) regions. PAM is highlighted in green.

To confirm that mutations had occurred in the *gch* and *tyr* genes in these mutant fish, the areas of exon-1 of these two genes were amplified by genomic PCR using individual embryos from the *gch/tyr* double mutant. The amplified DNA was subcloned into the pGMT Easy plasmid vector and sequenced. Sequenced DNA showed mutations occurred both in the *gch* and *tyr* genes in the expected region around the two-crRNA sequence in the exon. The mutations found in the *gch* showed small deletions both in the crRNA1 and crRNA2 regions (Fig. 3A). In contrast, mutations found in the *tyr* showed large deletion stretching from crRNA1 to crRNA2 sequence (Fig. 3B). Though the size of mutations was highly different between *gch* and *tyr* genes, we have confirmed specific deletion occurred in the designed exon (exon-1) of these genes causing frameshift mutations that are consistent with our observation of the loss of fluorescence and black pigments respectively (Fig. 2).

### Impact of UV on mutant embryo pigmentation patterns

**Fig. 4:**
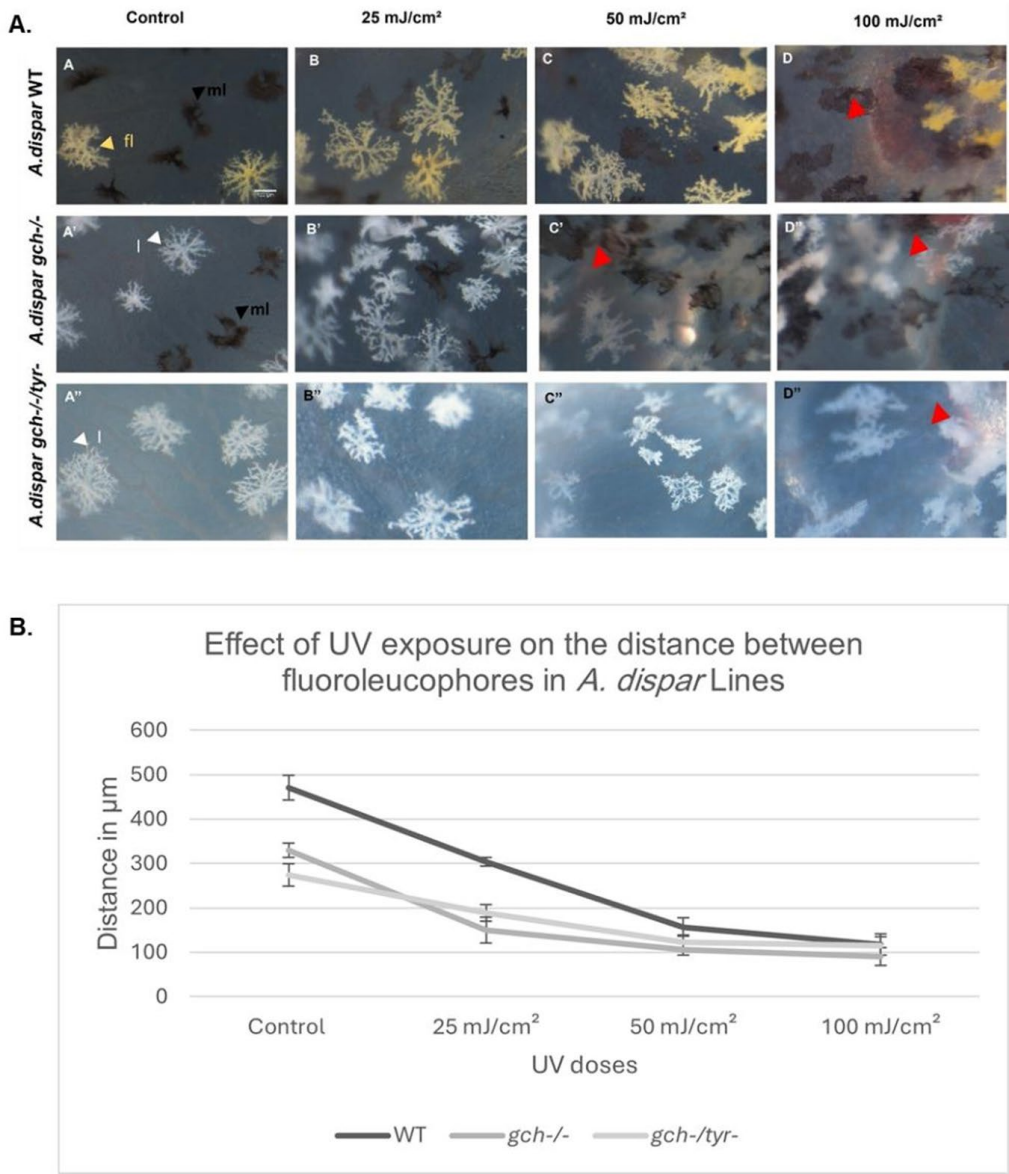
**A** Live image of the *A. dispar* embryos after exposure to UV. The embryos were exposed to 25, 50 or 100 mJ/cm^2^ UVC at 4dpf and imaged after 24 h post exposure with focusing on fluoroleucophores (yellow or white) on the surface of the yolk. a-d, WT, e-h. *gch-/-*, i-l *gch-/-tyr-/-*. Scale bar: 100 μm. **B** Effects of UV exposure on the distance between fluoroleucophores in the three lines of *A*. *dispar* WT, *gch-/-* and *gch-/-tyr-/-* after 24 h of UV exposure. Data are presented as mean ± SEM (*n* = 3). Post-hoc comparisons revealed a significant reduction in cell distances with increasing UV doses between control and UV-treated groups among lines (*p* < 0.001). WT exhibited the highest overall cell distances, followed by *gch-/-* and *gch-/-tyr-/-.*

To investigate the effects of UV light we exposed WT, *gch* and *gch/tyr* mutant embryos to UVC 254 nm light. Embryos were collected from these strains through natural spawning, incubated for 4 days at 28 °C and then exposed to UV at 4dpf with a variety of doses ranging from 25 to 100 mJ/cm^2^ (Fig. 4A). In embryos not exposed to UV, melanophores and fluoroleucophores typically maintained a distance of one to two cell diameters from each other. However, in embryos exposed to UV, the shape of these pigment cells was more often distorted, and they were positioned much closer to each other (Fig. 4A, B). The distance between fluoroleucophores was measured across the three *A*. *dispar* under using ImageJ software and compared to corresponding control distances within each line. The results showed a reduction in the distance between fluoroleucophores following UV treatment across all lines, indicating cell aggregation caused by the UV exposure (Fig. 4B). Besides the decrease of the distance of fluoroleucophore by UV exposure, the distance in the UV-unexposed embryos also showed differences between the WT and mutants; Wild type shows wider distance above 450 μm, and *gch-/-* and *gch-/-tyr-/-* showed shorter distance around 300 μm.

### Impact of UV on mutant embryo viability

**Fig. 5:**
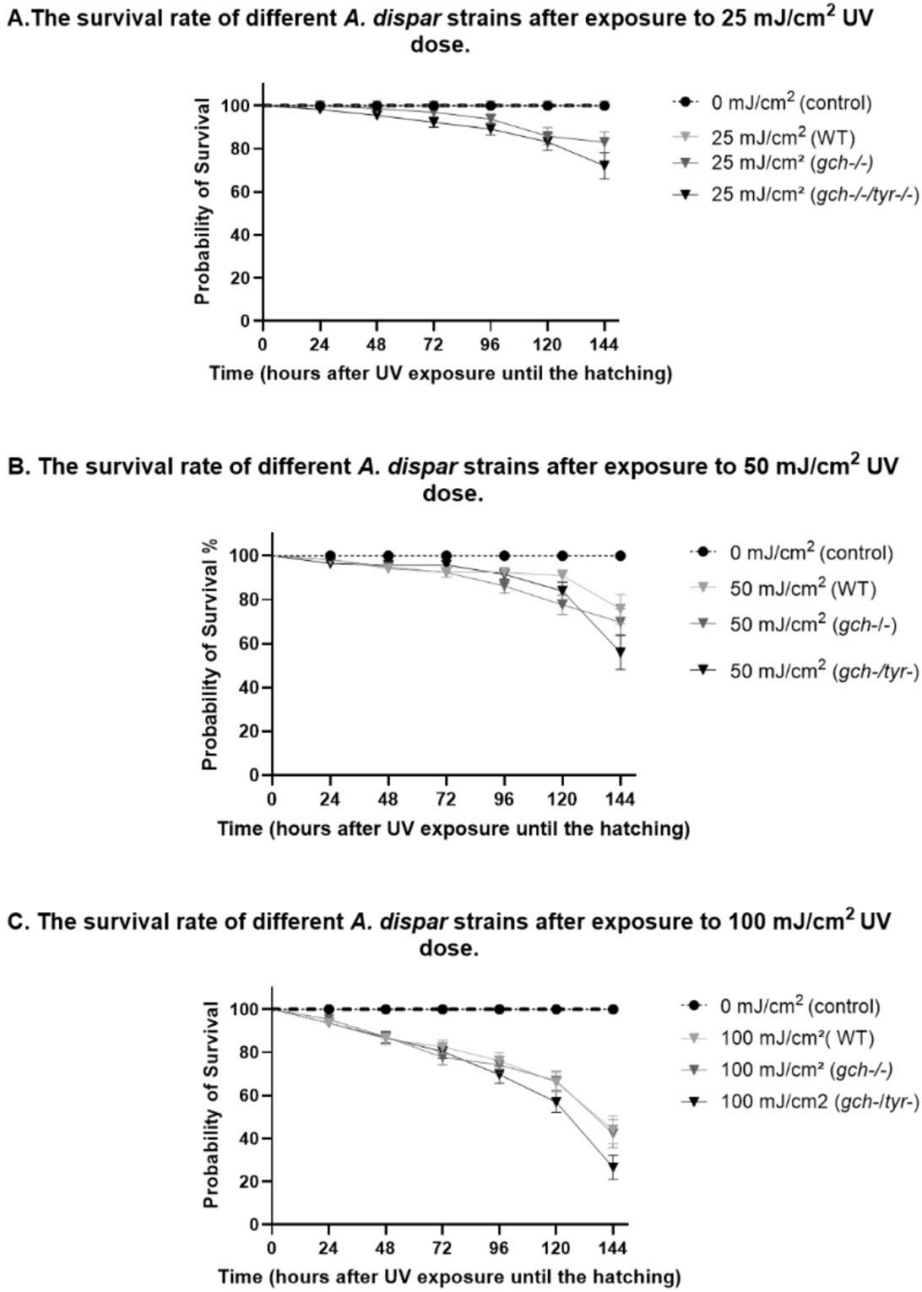
The combined analysis of survival rates across *A. dispar* lines WT, *gch*-/- and *gch-/-tyr-/-* under each dose of UV: A. at 25 mJ/cm^2^, 50 mJ/cm^2^, and100 mJ/cm^2^ compared to control 0 mJ/cm^2^, up to 144 h, *n* = 10. All *A*. *dispar* lines showed a reduction in survival in a dose-dependent manner at higher doses. While *A*. *dispar* WT line exhibited the highest resistance, *A*. *dispar gch-/-* and *gch-/-tyr-/-* showed a greater sensitivity, particularly double mutant *gch-/-tyr-/-.* Statistical analysis confirmed significant differences (*p* < 0.001) in survival rates between genotypes and UV doses, especially at the higher 100 mJ/cm^2^ dose.

The analysis of survival rates across A. *dispar* lines (WT, *gch-/-*, and *gch-/-tyr-/-*) under each UV dose (25, 50, and 100 mJ/cm^2^) revealed a dose-dependent reduction in survival rates over 144 h (Fig. 5). At the UV dose of 25 mJ/cm^2^, the mutant strains *gch-/-* and *gch-/-tyr-/-* exhibited a reduction in survival compared to unexposed control, while the wild-type showed no reduction in survival against non-exposed control embryos (Fig. 5A). At 50 mJ/cm^2^, survival decreased by UV in all strains with WT relatively mild lethality and *gch-/-tyr-/-* as highest lethality (Fig. 5B). At 100 mJ/cm^2^, all strains showed enhanced lethality but *gch-/-tyr-/-* consistently embryos showed highest lethality over WT and *gch-/-* (Fig. 5C). These findings highlight the critical role of pigmentation in UV resistance among *A. dispar* lines.

**Fig. 6:**
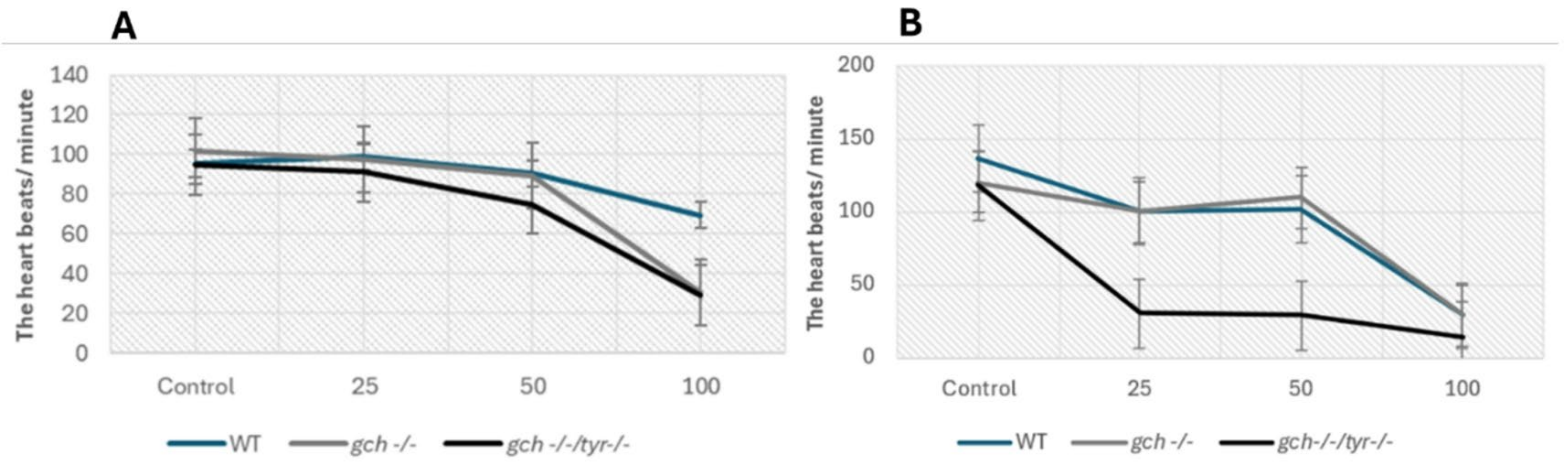
Heart rate is reduced by UV exposure depending on the genotype. Heart rate was measured after UV exposure in the WT, *gch* and *gch/tyr* embryos. **A** after 24 h post UV exposure. **B** after 48 h post UV exposure.

**Fig. 7:**
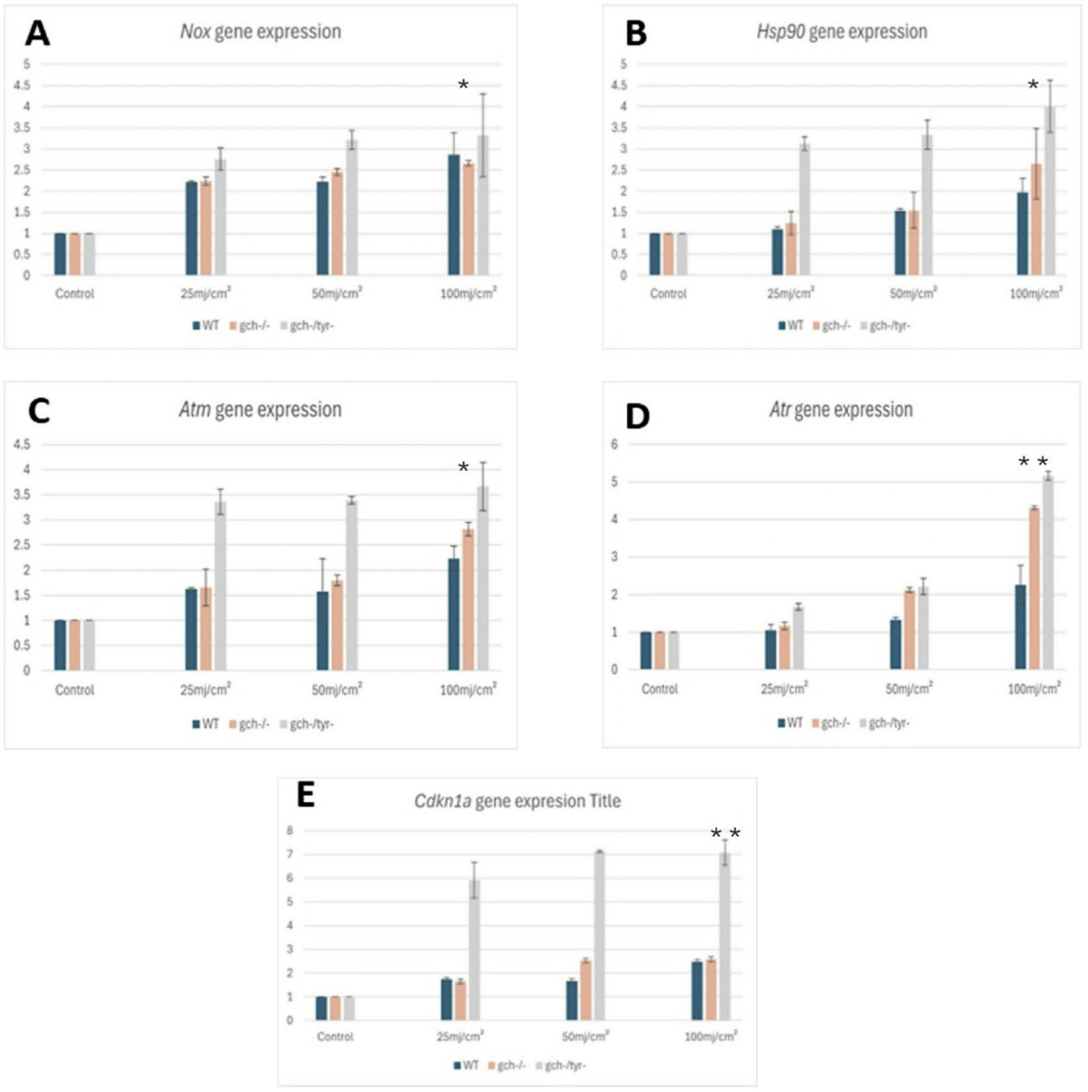
Quantitative PCR analysis of UV responding gene. **A** *nox.1*, **B** *hsp90*, **C** *atm*, **D** *atr* and **E** *cdkn1a* expression levels at 24 hp. Each experiment was performed in triplicate with *n* = 5 per experiment. Values plotted are means ± SEM. where significant differences are represented by * (*p* < 0.05) and ** (*P* < 0.01).

To examine the additional milder phenotypes of UV toxicity in the survived embryos from three genotypes, we have examined the heart rate of the UV-exposed embryos at 24 h and 48 post-exposures (Fig. 6). UV exposure decreased heart rate across all genotypes in a dose-dependent manner. At 24hpe, WT embryos exhibited the highest resistance to the UV, maintaining a higher heart rate (Fig. 7A), while *gch* and *gch/tyr* showed markedly reduced heart rates. In contrast, at 48 hpe, both WT and *gch* showed a similar dose-dependent decline in heart rate, with *gch*/*tyr* having a more severe decline, suggesting acceleration of the heat defect in the *gch* mutant compared to WT.

### UV-induced gene expression

To examine molecular and genetic mechanisms of UV mediated cellular stresses and damages, we have subsequently examined gene expression using qPCR. Total RNAs were extracted from UV exposed embryos with WT, *gch* and *gch/tyr* strains. Complementary DNA was synthesised and used for examining gene expression levels of oxidative response gene, *nox1*, heat response gene *hsp90*, and DNA damage repair genes *atm*, *atr*, cell cycle arrest gene *cdkn1*a and a house-keeping gene, *ef1α* as a reference gene. Following UV exposure, expression levels showed observable variations. Overall, elevated expression levels were observed across all genes tested after UV exposure, particularly among embryos exposed to 50 and 100 mJ/cm^2^ doses. Nevertheless, *gch*^*−*^*/*^*−*^*/tyr*^*−*^*/*^*−*^ exhibited the highest gene expression level compared to WT and *gch*^*−*^*/*^*−*^. Additionally, *gch*^*−*^*/*^*−*^ showed some elevated sensitivity to UV compared with WT (Fig. 7).

Although the overall trend is conserved, the dose responses in each gene showed some unique patterns. Firstly, *nox1* expression was upregulated by UV dose-dependent in all three strains, but there was no significant difference between WT and mutants except for a slight upregulation of the gene expression in the *gch/tyr* double mutant. In contrast, all other genes showed elevated responses in the pigment mutants against WT with varied patterns of sensitivity (Fig. 7A).

In contrast, the cell cycle arrest gene *cdkn1a* was the most prominent marker gene being highly induced in the *gch/tyr* double mutant from the lowest dose of UV, 25 mJ/cm^2^. However, this gene was not highly induced by UV in the WT and *gch* mutant (Fig. 7E).

The three remaining genes, *hsp90*,* atm* and *atr* exhibited the intermediate responses compared to the two genes mentioned above, suggesting that their expression is influenced by dose and both pigments dependent manner (Fig. 7B-D).

## Discussion

The Arabian killifish embryos are highly resistant to UV compared with the one in zebrafish. Zebrafish embryos all die at 10 mJ/cm2 UVC within 24 h but majority of Arabian killifish embryos survives with 25 mJ/cm2 UVC even after 6 days post exposure (Fig. 5A). With UVB (300 to 1200 mJ/cm2), zebrafish embryos still can show morphological deformity but in the Arabian killifish embryo, no clearly observable abnormality were observed (Kudoh et al. in preparation). Therefore, to assess the protective and differential role of pigments in this highly UV-resistant animal model, using UVC was the best assay system for the purpose. Effects of UV have been extensively studied in the cell culture and fish embryos both in the long wave length UV, UVA and UVB^23,33–36^ and in the short wave length UVC^26,28,31,36^. From these previous studies. From these studies, it has been known that the overall effects and toxicity pathway of UV are highly conserved in different UV lights with varied wavelengths, suggesting that UVC can be a useful and effective assay method for examining UV toxicity in cells and organisms. However, the intensity of toxicity are highly different between UVB and UVC depending on works: UVC shows more than 20 times higher damage to the cells^36^. The dose of UVC that was used in this experiment was very high compared to most of previous experiments done in the zebrafish and cell culture system, but it did not cause major lethality suggesting that the Arabian killifish embryo is highly resistant to UV possibly due to the habitat condition with strong sun light. It is also important to consider that long-wavelength UV can induce photo-repair. Because of this, long wavelength UV, or with a more realistic natural environment, a mixture of the middle to long wavelength and visual light has complicated cytotoxic and photo-repair effects. Therefore, it would be ideal to investigate the toxicity of UVA, UVB and mixed effects in following future works by using the assay methods and gene markers that we have characterised in this first report.

Our data demonstrated how pigment cells containing melanin and pteridine are essential in UV protection in fish embryos, and cells without such pigments become easily damaged by UV. The common deformities in fish embryos induced by UV are curvature or twisting of the spine^23^, reduction in growth and delay of hatching that possibly results from decreased metabolic rate similar to recorded responses in adult cichlid fish (*Cichlasoma nigrofasciatum*)^37^. Also, irritated embryos showed blood accumulation in their body, which might be a response to heartbeat changes or injury of blood vessels.

Svitačová et al. (2023)^38^ revealed that albino fish are more sensitive to UV stress than coloured fish in aquaculture, where pigmentation protects against ultraviolet (UV) radiation by absorbing UV radiation, mitigating genotoxic stress. Research on *Xiphophorus hybrids* by Funayama et al.. (1993)^39^ demonstrated that melanin pigmentation potentially offers protection, as darker fish exhibited reduced UV-induced DNA damage. In addition, albino olive flounders treated with UV had significantly lower survival rates than those not treated with UV^40^. As well as melanin also acts as an antioxidant and generates free radicals when exposed to UV light^41,42^.

In our results, the pigmented cells exhibited aggregation after UV exposure in the three lines and displayed more dendritic pattern in WT *A. dispar* than non-treated pigmented cells. Depending on the light intensity, aggregation of both melanophores and fluoroleucophores responses progressed^43^ and they were destroyed at high levels of UV doses. It is of interest that the distance between fluoroleucophores in *gch-/-* and *gch-/-tyr-/-* showed shorter distance than WT. This may also suggest importance of photoprotection by the pteridine pigment as lack of this pigment may have affected the cell number, size and/or distribution.

Previous studies have shown that ultraviolet (UV) radiation in fish can cause a range of potential effects, including direct DNA damage, oxidative stress, and phototoxicity^26,44,45^. To examine such cellular toxicities, we tested expression of genes related to oxidative stresses (*nox1*), protein damage (*hsp90*), and DNA damage (*atm*,* atr* and *cdkn1a*). All of these genes were induced by UV exposure in a dose dependent manner with varying patterns of response. *nox1* was the most markedly induced gene from the lowest dose of exposure (25 mJ/cm^2^). In addition, *nox1* is the only gene that exhibits a similar induction pattern in all three genetic strains tested. This can be explained by the known fact that *nox1* is induced by reactive oxygen species that is generated by UV. Since ROS production is a normal cellular process involved in cell maintenance, even at the lowest UV doses and regardless of pigment pattern, UV may induce a normal level of oxidative stress in all three genetic strains. On the other hand, other genes, such as *hsp90* is induced by the increase of denatured proteins while *atm*, *atr*, and *cdkn1a* are induced by DNA damage. This suggests that these genes are upregulated in response to cellular damage. Since pigment mutants had more damages in the cell compared to the WT, gene expression of these genes are more highly activated in the pigment mutants in a dose dependent manner. *gch* mutant exhibited slightly elevated gene expression responses compared to WT, while *gch/tyr* double mutant showed significantly higher responses in *hsp90*,* atm*,* atr* and *cdkn1a.* These findings suggest that both pteridine in the fluoroleucophore and melanin in the melanophore play important roles in protecting cells from UV.

For *nox1* and *cdkn1a*, the dose-response between WT and *gch* mutant was very similar. However, in the case of *hsp90*, *atm* and *atr*, the *gch* mutant exhibited an elevated response. This might be due to the mechanisms of UV protection by pteridine fluorescent pigment. Melanin can absorb light and, therefore, can generate heat, but pteridine emits light and does not generate heat when the cells are exposed to UV. Therefore, in the pteridine defective mutant, *gch*, heat stress can be induced which may lead to upregulation of *hsp90*. Besides *hsp90*,* atm* and *atr* showed elevated expression in the *gch* mutant compared with WT. This is also an interesting observation that suggests the important role of pteridine in protecting DNA from damage. One possibility is photoreactivation by fluorescent pigment, which could repair the DNA damage caused by UV. In the *gch* mutant, photoreactivation might be reduced, and DNA damage could be enhanced, causing enhanced expression of *atm* and *atr*.

*cdkn1a* exhibited the highest response to the lowest dose in the *gch/tyr* double mutant (6 folds increase compared to UV untreated control). In contrast, the gene response was quite mild in the WT and *gch* mutant. These findings also suggest that the mechanisms of UV protection by melanin/melanophore and pteridine/fluoroleucophore are distinct and may be related to the issue of heat or photoreactivation, as discussed above.

*cdkn1a/p21* is responsible for inhibiting cyclin-dependent kinases (CDKs), which regulate the progress of the cell cycle by phosphorylating target proteins. In addition, *p21* regulates transcription, apoptosis, DNA repair, and cell motility^46^. Therefore, strong activation of cdkn1a at the lowest dose in the *gch/tyr* double mutant highlights the importance of these pigment cells in maintaining the cell cycle and other cellular activities. Without these pigments, even a low dose of UV can suppress major cellular activities, such as cell proliferation.

Our data showed that pteridine can act as a biological photoprotection reagent. This may suggest potential technological applications of pteridine for eco-friendly sunscreen, or development of UV resistance organisms such as aquaculture species.

As a limitation of our study, we have focused on research using UVC as a potent analogue of longer wavelength UV (UVA and B). Unlike UVA and B, solar UVC does not reach to the ground level, therefore the experimental method that we have chosen can be consicdered artificial. Thought UVB and C showed similar effects in DNA damage, oxidative stress and other mechanisms, it might be possible that such an artificial system may induce unnatural effects to the fish embryos. To examine natural effects of UV in realistic conditions, UVA, B, C and a combination of different wavelength with various doses should be examined.

To conclude, we have discovered that both melanin and pteridine contribute in UVC protection in the Arabian killifish embryo. The gene response data suggest distinct mechanisms of UV protection between two pigment cells: *atm*, *atr* and *hsp90* more reliant on pteridine, while *cdkn1a* more reliant on melanin. In contrast, *nox1* is less dependent on these pigments, resulting in a similar response to UV in all three genetic strains. To fully elucidate the differential roles of pteridine and melanin, further investigations are needed using different wave length of UV with whole transcriptome analyses and molecular, cellular and biochemical analyses including cell cycle, apoptosis and DNA damage analyses.

## Supplementary Information

Below is the link to the electronic supplementary material.

Supplementary Material 1

## Data Availability

The datasets generated during the current study are available in the Genbank (accession numbers, PQ588425 for *gch* and PQ588426 for *tyr, * respectively).
