# Supplementary material for: Melanophore and fluoroleucophore photo-protect the Arabian killifish, Aphanius dispar, embryo from ultraviolet light
Source: Sci Rep. 2026 Feb 3;16:7091. doi: 10.1038/s41598-026-37311-6 (PMC12920782; doi:10.1038/s41598-026-37311-6)
Supplement: Supplementary file 1 — Supplementary Material 1 [file 41598_2026_37311_MOESM1_ESM.pdf]

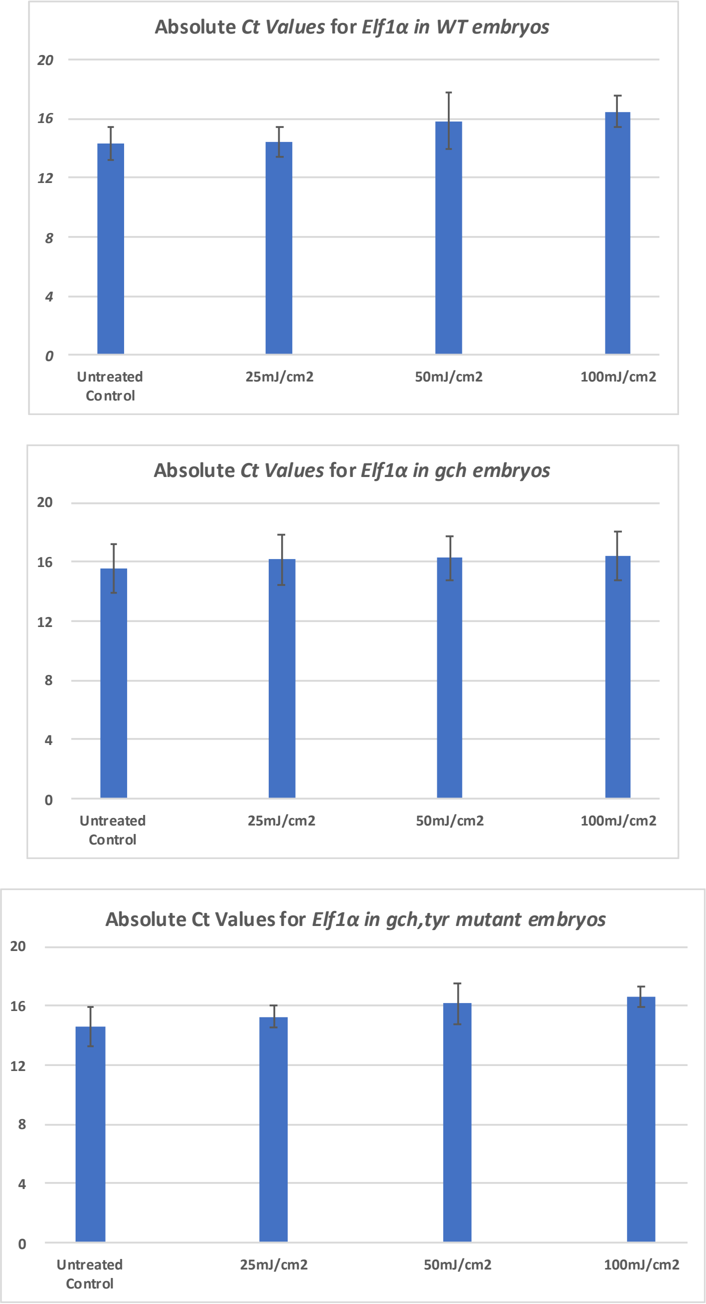

Figure S1. Absolute Ct value for the reference gene, *EF1alpha*, analysed by qPCR in the WT, gch-/- and gch-/-/tyr-/- embryos treated with UV.
